# Antibacterial activity of calcium hydroxide combined with chitosan solutions and the outcomes on the bond strength of RealSeal sealer to radicular dentin

**DOI:** 10.7555/JBR.26.20110136

**Published:** 2012-04-29

**Authors:** Shaymaa Elsayed Elsaka, Amr Mohamed Elnaghy

**Affiliations:** a Department of Dental Biomaterials, Faculty of Dentistry, Mansoura University, Mansoura, PC 35516, Egypt;; b Department of Conservative Dentistry and Endodontics, Faculty of Dentistry, Mansoura University, Mansoura, PC 35516, Egypt.

**Keywords:** antibacterial, calcium hydroxide, chitosan, *Enterococcus faecalis*, push-out, RealSeal

## Abstract

The purpose of this study was to investigate the antibacterial activity of calcium hydroxide [Ca(OH)_2_] combined with chitosan solutions against *Enterococcus faecalis*-infected root canal dentin and the effect of this new intracanal medicament on the bond strength of RealSeal sealer to radicular dentin. An experimental intracanal medicament was prepared by mixing different concentrations of chitosan solution (25%, 50%, and 100%, *W/V*) to Ca(OH)_2_ powder. Antibacterial activity was evaluated and the total numbers of colony forming units were determined. Bonding ability of RealSeal sealer to radicular dentin was evaluated using push-out bond strength test. Data were analyzed using one-way analysis of variance (ANOVA) and Tukey's multiple comparison tests. We found that Ca(OH)_2_ combined with different concentrations of chitosan solutions showed better antibacterial activity than Ca(OH)_2_ mixed with saline, without significantly affecting the bond strength of RealSeal sealer to radicular dentin (*P* > 0.05). The findings suggest that Ca(OH)_2_ combined with chitosan is a promising intracanal medicament and may be effective in endodontic therapy.

## INTRODUCTION

Bacteria are the major contributing factors in the development of pulpal and periapical inflammation[1]. Consequently, elimination of bacteria from the infected root canal system is the main objective of endodontic treatment in order to prevent reinfection[2]. Anticipated long-standing success of root canal treatment requires efficient debridement and disinfection of the root canal system[3]. Long-lasting endodontic infection might be attributed to the bacteria in the entire root canal system, including isthmi, ramifications, fins and dentinal tubules[4]. One of the virulence factors contributing to the persistence of these bacteria is their ability to form biofilms[5], particularly in medically at risk patients. Bacteria in these regions are frequently not affected by chemomechanical preparation and thus jeopardize the outcome of root canal treatment[6]. On the other hand, before root canal obturation, chemomechanical preparation combined with appropriate antimicrobial intracanal medicament for adequate time can be more efficient in elimination of bacteria[7].

*Enterococcus faecalis* (*E. faecalis*) is a bacterium commonly found in endodontic infections, particularly in retreatment cases. *E. faecalis* can be very easily eliminated in planktonic forms *in vitro*. However, it appears to turn out to be more resistant while it is present in an infected root canal system. The resistance may be caused by the activation of virulence factors, biofilm formation, or invasion of dentinal tubules. While *Enterococci* have both intrinsic and acquired resistance to many antibiotics, they are inherently more resistant to antimicrobial drugs than other clinically significant Gram-positive bacteria[8],[9]. Since most endodontic research focuses on the eradication of this bacterium[5], *E. faecalis* was used in this current study.

Calcium hydroxide [Ca(OH)_2_] has been widely used in endodontics as an intracanal medicament. The formulations of Ca(OH)_2_ with high pH have damaging effects such as inactivation of the membrane transport system on the biological properties of bacterial cell walls, resulting in bacterial toxicity[10]. Nevertheless, *E. faecalis* has been found to be resistant to this effect due to its capability to go through the dentinal tubules and adapt to altering environments[11]. Various antibacterial agents have been introduced, such as chlorohexidine, metronidazole, bioactive glass, or their combination with Ca(OH)_2_, which were found to be more efficient than Ca(OH)_2_ alone in minimizing the number of *E. faecalis*, with varying degrees of success[12],[13]. Consequently, the discovery and development of the medicaments to control the infection of root canal is urgently needed.

Chitosan (CS) is a naturally occurring polysaccharide biopolymer that is produced by alkaline partial deacetylation of chitin. Chitin is a straight homopolymer consisting of (1,4)-linked *N*-acetyl-glucosamine units, which can be found in the exoskeleton of crustaceans such as crabs and shrimps. CS is composed of copolymers of glucosamine and *N*-acetyl-glucosamine. CS is generally regarded as biocompatible, non-toxic, and biodegradable and is inherently antibacterial in nature[14],[15].

In recent years, methacrylate resin-based sealers have been developed on the basis of dentin adhesion strategies in order to seal the root canal more effectively and strengthen the root structure[16]. Long-term sealing ability and adaptation to the root canal walls are one of the prime fundamentals for a root canal sealer[17]. RealSeal system (SybronEndo, Orange, CA, USA) has been introduced as an alternative to guttapercha and conventional sealers. This new system has been reported to have excellent sealing ability[18]; however, it has no antibacterial activity[19],[20]. The use of intracanal medicaments should not jeopardize the bond strength of obturating materials to radicular dentin[21]. Therefore, the aim of this study was to evaluate the antibacterial activity of Ca(OH)_2_ combined with CS as an intracanal medicament and the effect of this new intracanal medicament on the bond strength of RealSeal sealer to radicular dentin.

## MATERIALS AND METHODS

### Preparation of CS solutions

Different concentrations of CS ( 85% deacetylated, Sigma-Aldrich, St Louis, MO, USA) solutions were prepared in 1 L of 1% (*V/V*) acetic acid[14] to obtain different vehicles of CS solution to be mixed with Ca(OH)_2_ powder (Sigma-Aldrich) (***Table 1***).

**Table 1:** Different concentrations of chitosan (CS) solution used in this study

Group	CS concentration [g/L of 1% (*V/V*) acetic acid]
Saline (control)	0
Ca(OH)_2_-saline	0
Ca(OH)_2_-25% CS	0.5 (25 *V/V*, %)
Ca(OH)_2_-50% CS	1.0 (50 *V/V*, %)
Ca(OH)_2_-100% CS	2.0 (100 *V/V*, %)

### Preparation of dentin specimens

The method used was a modification of the one previously described by Haapasalo and Ørstavik[22]. One-hundred-and-fifty single-rooted human teeth, extracted for orthodontic or periodontal reasons, were selected. They were obtained by protocols that were reviewed and approved by the institutional review board of the Faculty of Medicine and Dentistry, Mansoura University, Mansoura, Egypt. The crown and the apical part of the root were removed with a low speed diamond saw (Isomet 1000, Beuhler Ltd., Lake Bluff, IL, USA) under water-cooling to obtain 6 mm of the middle third of the root. The root cementum was not removed from the root surface. Standardization of root canal diameters was performed by using Gates Glidden drills, No. 3 (Dentsply Maillefer, Ballaigues, Switzerland) in a slowspeed handpiece. The specimens were placed in an ultrasonic bath of 17% ethylenediaminetetraacetic acid (EDTA) for 4 min followed by 3% sodium hypochlorite (NaOCl) for 4 min to remove organic and inorganic debris. Trace chemicals were removed by immersing the dentin specimens in an ultrasonic bath of distilled water for 5 min and then autoclaving for 20 min at 121°C[12],[23].

### Contamination of dentin specimens with *E. faecalis*

The test organism used for this study was *E. faecalis* (ATCC 29212), which was grown in tryptone soya agar (Oxoid Ltd., Basingstoke, UK) for 24 h. Each dentin block was placed in pre-sterilized microcentrifuge tubes containing 2 mL tryptone soya broth (Oxoid Ltd.) with 1×10^7^ colony-forming units per milliliter (CFU/mL) of a 24-h old *E. faecalis* suspension. All procedures were performed under a laminar flow hood (NuAire, Plymouth, MN, USA). Purity of the culture was checked regularly. Contamination of the specimens was carried out for a period of 21 d[12].

### Assessment of antibacterial activity

Following the contamination period, the specimens were irrigated with sterile saline, dried with sterile paper points, and divided into five groups (*n* = 30 dentin blocks) according to the types of intracanal dressings: Group 1: saline (negative control); Group 2: Ca(OH)_2_ was mixed with sterile saline in a ratio of 1.5:1 (*W/V*) to obtain a paste like consistency[12]. From Group 3 to Group 5, Ca(OH)_2_ powder was mixed with different concentrations of CS solutions as presented in ***Table 1***.

The medicaments were placed inside the canals which were then sealed coronally and apically with Cavit (3M ESPE, Seefeld, Germany). The specimens were placed into Petri dishes and covered with humid sterile gauzes and incubated at 37°C for 1, 7, and 14 d. The root canals were then irrigated with sterile saline and dried with sterile paper points. Harvesting of dentin was performed at two depths (200 and 400 µm) with Gates Glidden drills No. 4 and 5, respectively, with 10 specimens at each time interval. The collected dentin chips were transferred into 1 mL sterile tryptone soya broth and incubated at 37°C for 24 h. After the incubation period, the content of each tube was serially diluted, five times, with 100 µL broth in 100 µL sterile saline. Fifty microliters of the dilution was then plated on tryptone soya agar plates and incubated for 24 h[12]. Colonies were counted and the total CFU was calculated; the values were then converted to their log_10_ values.

### Push-out bond strength

#### Specimen preparation

One-hundred extracted, single-rooted human teeth were used to evaluate bond strength. After establishment of canal patency, the root canals were mechanically prepared by using ProFile nickel-titanium rotary instruments (Dentsply Maillefer) in a crown-down technique, under constant irrigation with 3% NaOCl. Each canal was prepared to ISO size 30, 0.06 taper and the working length was established 1 mm short of the apex. The root canals were then irrigated with 17% EDTA. Finally, the roots were irrigated with 10 mL distilled water to avoid the prolonged effect of EDTA and NaOCl solutions. The debrided root canals were dried with multiple paper points and randomly divided into five groups (*n* = 20/group) on the basis of CS concentration as a vehicle for Ca(OH)_2_ intracanal dressing (***Table 1***). Then, the medicaments were placed inside the canals and sealed coronally and apically with Cavit and stored in 100% humidity at 37°C for 7 and 14 d (*n* = 10/group).

#### Canal filling

After the incubation period, the root canals were irrigated with 3% NaOCl, 17% EDTA, and finally with 10 mL of distilled water. The canals were then dried with paper points. RealSeal self-etching primer (SybronEndo, Orange, CA, USA) was introduced into the canal with a microbrush and allowed to remain for 30 s; the excess was removed with paper points. RealSeal sealer was then introduced along the entire length of the canal using a paper point. A Resilon point, previously tried-in with tug back, was coated with RealSeal sealer and inserted to the working length. After that, the Resilon point was compacted using a warm vertical compaction technique with a System B (SybronEndo) at 150°C. Backfilling was achieved with Obtura II (Spartan, Fenton, MO, USA) at 140°C. Then, the coronal surface of the root filling was light-cured for 40 s with a Coltolux LED curing light (Coltene Whaledent, Cuyahoga Falls, OH, USA) to accomplish an immediate coronal seal. The specimens were stored at 37°C and 100% humidity for 1 week to allow the sealer to set completely.

#### Push-out assessment

Each root was horizontally sectioned into five 1±0.1 mm thick serial slices (*n* = 5) by using a low speed diamond saw under water. After measuring the thickness of each slice with a digital caliper (Mitutoyo, Tokyo, Japan), the filling material was loaded with a 0.5 mm diameter cylindrical plunger. Loading was performed on a universal testing machine (Model TTB, Instron Corp., Canton, MA, USA) at a cross-head speed of 0.5 mm/min until debonding occurred. The load at failure recorded in newtons (N) was divided by the area of the bonded interface for each specimen to calculate the bond strength in megapascals (MPa), according to the following formula[24]: 
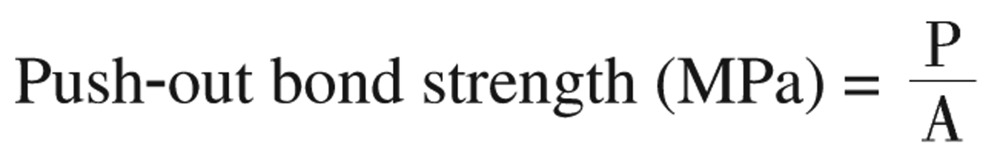
 where *P* is the maximum load (N) and A is the adhesion area of root canal filling (mm^2^).

The adhesion area of each section was calculated using the following formula:

*Adhesion area = L* × [π*r*_1_ + π*r*_2_]

*L* was calculated as follows:

*L* = [*h*^2^ + (*r*_1_ + *r*_2_)^2^]^1/2^

where *r*_2_ is the coronal radius, *r*_1_ is the apical radius, and *h* is the thickness of the slice. Debonded specimens were examined under a stereomicroscope (Olympus SZX-ILLB100-Olympus Optical Co. Ltd., Tokyo, Japan) at 50×magnification to evaluate the fracture pattern. The modes of failure were classified as adhesive failure along the sealer-dentin interface, cohesive failure within the sealer or mixed failure.

### Statistical analysis

The data were present as mean±SD, and one-way analysis of variance (ANOVA) and Tukey's multiple comparison tests were performed. The paired *t*-test was used to check differences at the depths and differences in growth at different time intervals within groups. Statistical significance was set at the 0.05 probability level.

## RESULTS

### Antibacterial activity

**Fig. 1:**
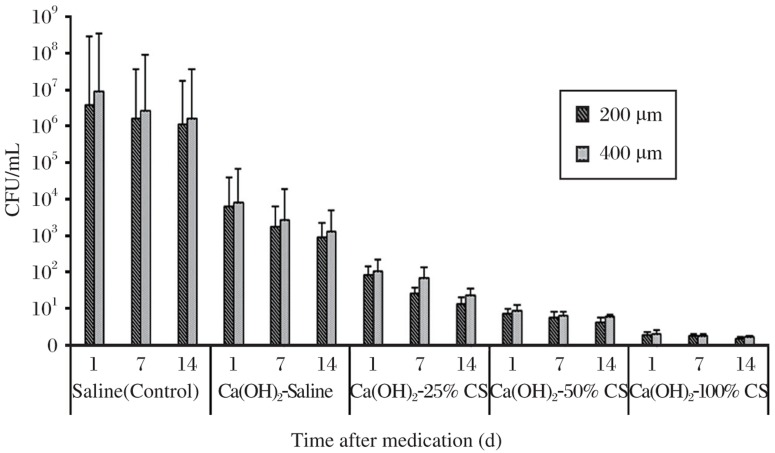
Mean log_10_ colony forming units (CFU) for different intracanal medicaments at 200 µm and 400 µm depths at d 1, 7, or 14. Ca(OH)_2_ combined with chitosan solutions were more effective in inhibiting the growth of *E. faecalis* compared with Ca(OH)_2_ mixed with saline.

The mean log_10_ CFU values and standard deviations for the different intracanal medicaments groups are presented in ***Fig. 1***. Ca(OH)_2_ mixed with CS solutions caused a significant reduction in the mean log_10_ CFU counts in comparison with saline (control) and Ca(OH)_2_ mixed with saline (*P* < 0.05). There was no significant difference between the growth inhibition of *E. faecalis* at 200 µm and 400 µm (*P* > 0.05). The Ca(OH)_2_-100% CS group was the most effective medicament against the tested bacteria in comparison with the other groups. No significant difference was found for bacteria counts at the three time intervals within the same group (*P* > 0.05).

### Push-out bond strength

**Fig. 2:**
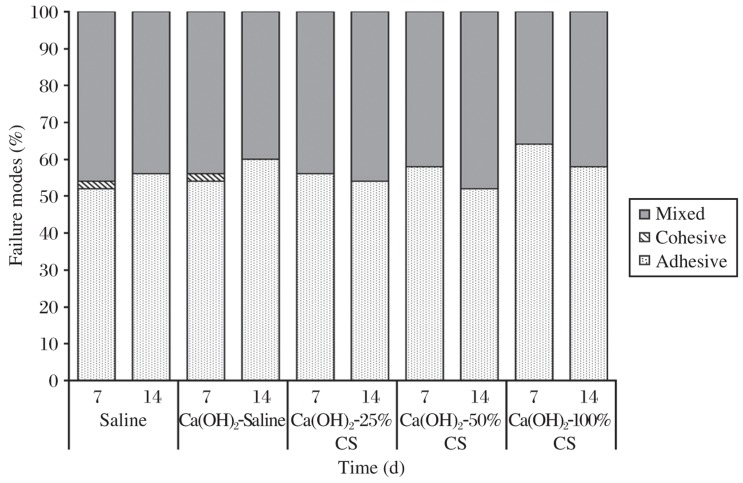
Failure pattern distribution of different groups tested. Most failure modes were adhesive for all groups.

The mean push-out bond strength (MPa) and standard deviations of RealSeal system to radicular dentin after removal of different intracanal medicaments at either d 7 or 14 are presented in ***Table 2***. There was no significant difference in the bond strength values among different groups (*P* > 0.05). Moreover, there was an insignificant reduction in the bond strength values in comparison with the control group at the two time intervals (*P* > 0.05, ***Table 2***). Most failure modes were adhesive for all groups (***Fig. 2***).

**Table 2:** Mean push-out bond strength (MPa) of RealSeal sealer to radicular dentin after removal of different intracanal medicaments at either d 7 or 14

Group	Day 7	Day 14
Saline (control)	0.58 + 0.26	0.52 + 0.21
Ca(OH)_2_-saline	0.43 + 0.18	0.36 + 0.12
Ca(OH)_2_-25% CS	0.46 + 0.16	0.43 + 0.13
Ca(OH)_2_-50% CS	0.48 + 0.12	0.44 + 0.16
Ca(OH)_2_-100% CS	0.45 + 0.16	0.40 + 0.12

CS: chitosan.

(mean±SD)

## DISCUSSION

Utilizing a biocompatible intracanal medicament with antibacterial properties between appointments may diminish, or eradicate bacteria, in the root canal system and considerably enhance the success of root canal therapy[25]. *E. faecalis* was chosen as the test organism as it is commonly found in infected root canals[26]. *E. faecalis* produced biofilm in root canals although intracanal dressing with Ca(OH)_2_[27]. This biofilm phenotype may be another factor contributing to the resistance of *E. faecalis* to most antimicrobial agents[28]. The potential role of *E. faecalis* in the failure of root canal therapy makes it essential to develop strategies to control infections attributable to this organism[29]. The major objective of this study was to test whether the combination of Ca(OH)_2_ and CS has a potential additive or synergistic effect on the viability of *E. faecalis*, an important endodontic pathogen.

The present study evaluated the antibacterial activity of Ca(OH)_2_ combined with CS solutions against *E. faecalis* using a modified method from that proposed by Haapasalo and Ørstavik[22]. Human permanent teeth were used instead of the bovine teeth suggested by Basrani *et al*[30],[31]. The canal lumens of the bovine blocks were 3 times larger than those of human blocks and can influence the antimicrobial activity of certain medicaments accordingly[31]. Furthermore, studies with human dentin blocks would certainly be more reliable to simulate the clinical scenario[12].

Several studies have revealed the limited ability of Ca(OH)_2_ to entirely eradicate bacterial cells inside dentinal tubules[22],[25],[32]. The low solubility and diffusibility of Ca(OH)_2_ as well as the dentin buffering ability reduced the antibacterial effect of Ca(OH)_2_[10],[23]. Most studies using the “infected tooth” model have showed that Ca(OH)_2_ is ineffective against E. faecalis, particularly when it is used alone without combination with any other antimicrobial agents[7],[22],[32],[33]. This could explain the minimal antibacterial effect of Ca(OH)_2_ compared with Ca(OH)_2_ mixed with different concentrations of CS solutions at the three tested time intervals (1, 7, and 14 d) with the two different depths (200 and 400 µm) in this study. It could be explained by the polycationic properties of CS, presented by the positively charged -NH_3_^+^ groups of glucosamine, which could be the major factor contributing to its interaction with negatively charged surface components of bacteria, resulting in extensive cell surface alterations, leakage of intracellular substances and ultimately causing damage of vital bacterial activities[15]. Consequently, it is possible that Ca(OH)_2_ combined with CS inhibits the growth of E. faecalis and subsequently it may inhibit bacterial re-entry and recolonization. A synergistic antibacterial effect was found in Ca(OH)_2_ combined with CS against E. faecalis when compared to Ca(OH)_2_ mixed with saline. The results revealed significant information on the more effective intracanal medicaments against E. faecalis.

Optimum adhesion requires intimate contact between the adhesive material and the substrate to facilitate molecular interaction and allow either chemical adhesion or penetration for micromechanical surface interlocking[34]. Adhesion of an endodontic sealer is defined as its capacity to adhere to the root canal walls and the ability to promote the union of the guttapercha cones to each other and to dentin[35],[36]. This concept can be applied to the filling systems that use different solid materials combined with the root canal sealer[35]. The bond strength of root canal sealers to radicular dentin is essential for preserving the integrity of the seal in the root canal filling[37]. The recent introduction of RealSeal system as an alternative root canal filling material is based on formation of a single resin block (monoblock), in which the core material, sealing agent and root canal dentin form a single cohesive unit that adheres to the root canal walls[38].

Bond strength testing has become a popular method for determining the effectiveness of adhesion between endodontic materials and tooth structure. There are various methods for measuring the adhesion of endodontic root canal sealers. However, none have yet been widely accepted[16],[38]. The tensile strength test is sensitive as small alterations in the specimen or in stress distribution during load application have substantial influence on the results[39]. On the other hand, a major problem with shear testing is that it is difficult to closely align the shear-loading device with the bond interface. The load is offset at some distance from the bonded interface, resulting in unpredictable torque loading on the specimen[40].

The push-out test is a reliable and efficient method to evaluate bond strength because it allows measurement of regional differences in bond strength along the root levels with adequate variability of the data distribution[37]. Another advantage of this method is that it allows root canal sealers to be evaluated even when bond strengths are low[38]. Accordingly, evaluation of push-out bond strength of the RealSeal sealer to radicular dentin was considered to investigate the effect of the newly experimental intracanal medicament on the radicular dentin bond strength.

Ca(OH)_2_ combined with CS solution was found to be advantageous, since the bond strength of RealSeal sealer to radicular dentin was not significantly different from that of the control. A slight insignificant decrease in the bond strength values was found in comparison with the control group at the two time intervals (7 and 14 d). This could be explained because Ca(OH)_2_ pastes are not easily removed from the root canal system, suggesting that Ca(OH)_2_ paste left into the root canal space could have interfered with adhesion of RealSeal sealer to radicular dentin[41],[42].

This study highlights the possible potential consideration of CS solution as a significant vehicle with Ca(OH)_2_ as intracanal medicament to ensure long-term success of endodontic treatment, suggesting a promising use of CS in resisting bacterial growth in the radicular dentin. Further laboratory and clinical investigations are required to examine the effect of Ca(OH)_2_ combined with CS as an intracanal medicament.

In conclusion, based on the results presented, and within the limitations of this study, the following conclusions can be made: Ca(OH)_2_ intracanal medicament incorporating CS solution as a vehicle exhibited an inhibitory effect on the growth of *E. faecalis* in the radicular dentin, compared to Ca(OH)_2_ mixed with saline, and Ca(OH)_2_ incorporating CS is a promising antibacterial intracanal medicament without adversely affecting the adhesive properties of RealSeal sealer to radicular dentin.
